# Thermo-Mechanical Characterisations of Flax Fibre and Thermoplastic Resin Composites during Manufacturing

**DOI:** 10.3390/polym10101139

**Published:** 2018-10-12

**Authors:** Shenglei Xiao, Peng Wang, Damien Soulat, Hang Gao

**Affiliations:** 1 School of Mechanical Engineering, Dalian University of Technology, Dalian 116000, China; shenglei.xiao@ensait.fr (S.X.); gaohang@dlut.edu.cn (H.G.); 2 ENSAIT, GEMTEX, University of Lille, F-59056 Roubaix, France; damien.soulat@ensait.fr

**Keywords:** textile composites, natural fibres, thermoplastic, thermo-mechanical behaviour

## Abstract

The flax fibre reinforced composites with advanced structure, which can be regarded as recyclable parts, are potential and promising materials in the automobile industry. During their manufacturing, the reinforcements or prepregs should be performed to the desired shape beforehand. Mechanical behaviours accordingly play an important role during this process. However, this preforming process is usually under high temperatures, thus, the mechanical behaviours could be modified under this state. Especially for reinforcements produced by flax yarns, has barely been studied. To fill this gap, in this paper the thermos-mechanical characterization of Flax/Polyamide12 (PA12) commingled yarn and prepreg woven fabric is analysed using tensile and in-plane shearing tests under different temperatures and tensile speeds. The results conclusively show that strength can be improved by increasing the temperature below the PA12 melting value on woven fabrics, which is inverse tendency for single yarn. Moreover, increasing tensile speed could increase the strength of the single yarn and fabric. This reveals that the PA12 fluidity has great influence on tensile behaviour. The characterisation results would be employed as prescriptive recommendations in the process of manufacturing flax fibre-reinforced composite parts.

## 1. Introduction

Thermoforming prepregs is a frequent process to manufacture recyclable composite parts in the automobile and aerospace industries. Recently, textile reinforcements made by natural fibres have the potential to be used in these industries due to their lightweight properties [1,2]. The mechanical properties of natural fibres, in particular those of flax, can be considered as a good choice for the reinforcement of composites due to their high stiffness, strength, and low density [3,4,5,6]. Thus, flax fibre-reinforced composites have gained more and more attention among researchers working in a broad range of domains [7,8,9,10]. Fundamentally, the mechanical behaviours of fabrics or prepregs are primary properties. Kayode et al. [11] investigate the impact and flexural properties of flax fabrics in terms of architectures and outer ply thickness. They find that composites manufactured by plain weave reinforcement have the best mechanical properties. On the other hand, during the composites manufacturing with advanced structure, the preforming process is quite important, and is heavily related to these mechanical behaviours. The deformability properties based on mechanical behaviour have been widely explored with respect to defect characterisation and different shapes [2,4,5]. Compared to investigations on deformability properties, studies of the mechanical behaviour of the fabric are scarce, especially for thermos-mechanical behaviours that play important role in thermoforming process.

Recently, the commingled fibre used to manufacture fabric could not only consolidate the structure to accentuate the mechanical properties, but also change its thermo-mechanical characteristics. For example, Wang et al. [12] experimentally investigated the thermo-mechanical behaviour of woven carbon/polyphenylene sulphide and polyetheretherketone prepregs based on bias-extension tests. This indicated that the temperature and related change in shear behaviour have strong consequences on the final composite parts. Similarly, Stretch Broken Carbon Fibre/PPS and PEEK commingled prepregs are also explored at temperatures to obtain different thermo-mechanical behaviour [13]. Compared to flax fibre, the Polyamide (PA12) twining around the fibre yarn to present the mixed properties of Flax and PA12 is usually capable of serving as reinforcements [14]. Although Xiao et al. [15] thoroughly discuss the in-plane shear behaviour of Flax/PA12 fabrics, the temperature during manufacturing can also affect the mechanical behaviour. Notably, the shear angle and its relationship to other conditions, such as force and deformation, is responsible for producing a simulated, reproducible curved form. This will allow us to accurately determine how the textile material performs under a range of temperature conditions, as the temperature is not generally constant during the thermoforming stage [3,12,13]. 

Therefore, the main objective of this paper is to characterise the tensile and in-plane shear thermo-mechanical behaviour of Flax/PA12 prepregs under varying temperatures. Before that, the single fibre is studied. These properties will allow us to accurately simulate the thermoforming process in order to utilise the material to the maximum of its potential in comparison to its composite counterparts.

## 2. Materials and Methods

### 2.1. Materials

**Figure 1:**
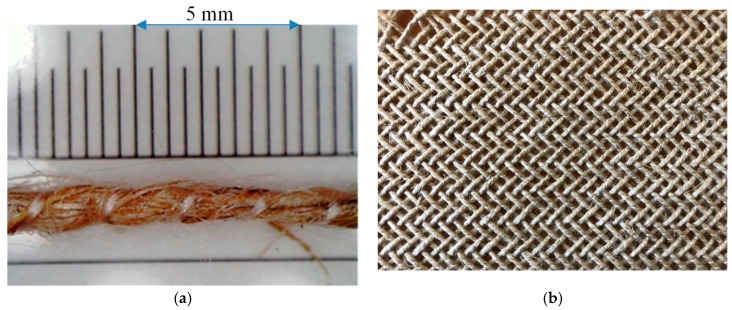
(**a**) Microscopic picture of Flax/PA12 commingled yarn and (**b**) Structure of 2-2 twill with Flax/PA12 commingled yarn.

The tested material is Flax/PA12 commingled yarn; the main properties are noted in Table 1. The mass fraction of flax and polyamide fibres is 64% and 36% respectively. An in-plane shear characterisation was performed for the woven fabric made by the commingled yarns mentioned above. Table 1 also shows the characteristics of the Flax/PA12 woven fabric; it can be noted that the woven fabrics have a well-balanced structure, as the amount of yarn per centimetre is identical in both warp and weft directions. Figure 1 displays the specific characteristics of the single yarn and woven fabric.

### 2.2. Methods

#### 2.2.1. Tensile Test

**Figure 2:**
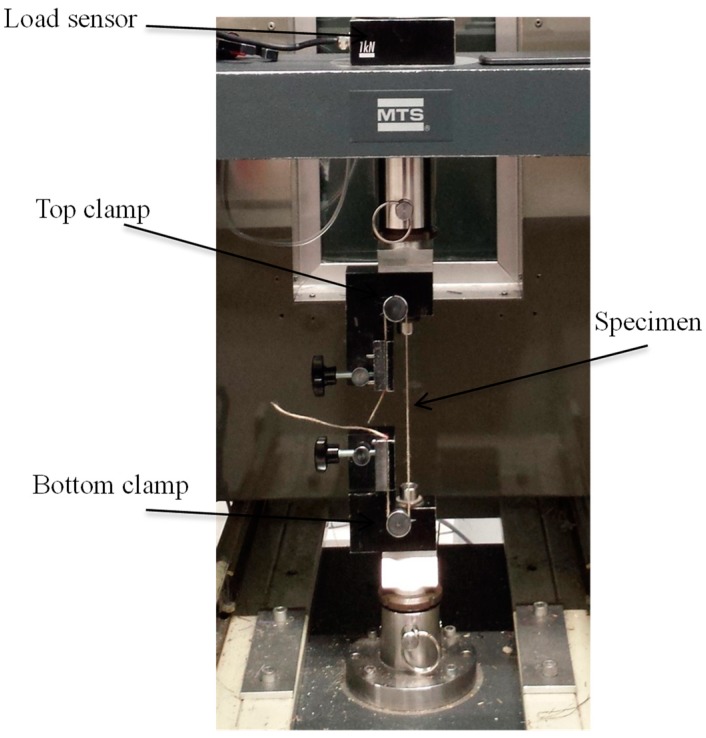
Set-up of the tensile machine with a single yarn specimen at ambient temperature.

Tensile tests were conducted using a universal tensile tester and an isothermal oven; Figure 2 shows the set-up of the machine. A single Flax/PA12 yarn was inserted and clamped by a top movable clamp. During the tests, a load sensor on the top clamp measured the force in real time. In order to ensure an accurate fixed length of the specimen, the machine was set at 150 mm. A preliminary test was conducted to verify that the slip of the test specimen was 0%, in order to obtain accurate results. In order to reach thorough conclusions about the performance of Flax/PA12 single yarn and its woven fabric, the tensile speed (extension rate) and temperature were systematically altered. Table 2 displays the details of the tests that can be divided into two parts to explore the influence of tensile speed and temperature in single Flax/PA12 yarn. It should be noted that the melting value of PA12 is 178 °C; the temperatures above and below this value were selected below in the tests.

#### 2.2.2. Bias Extension Test

**Figure 3:**
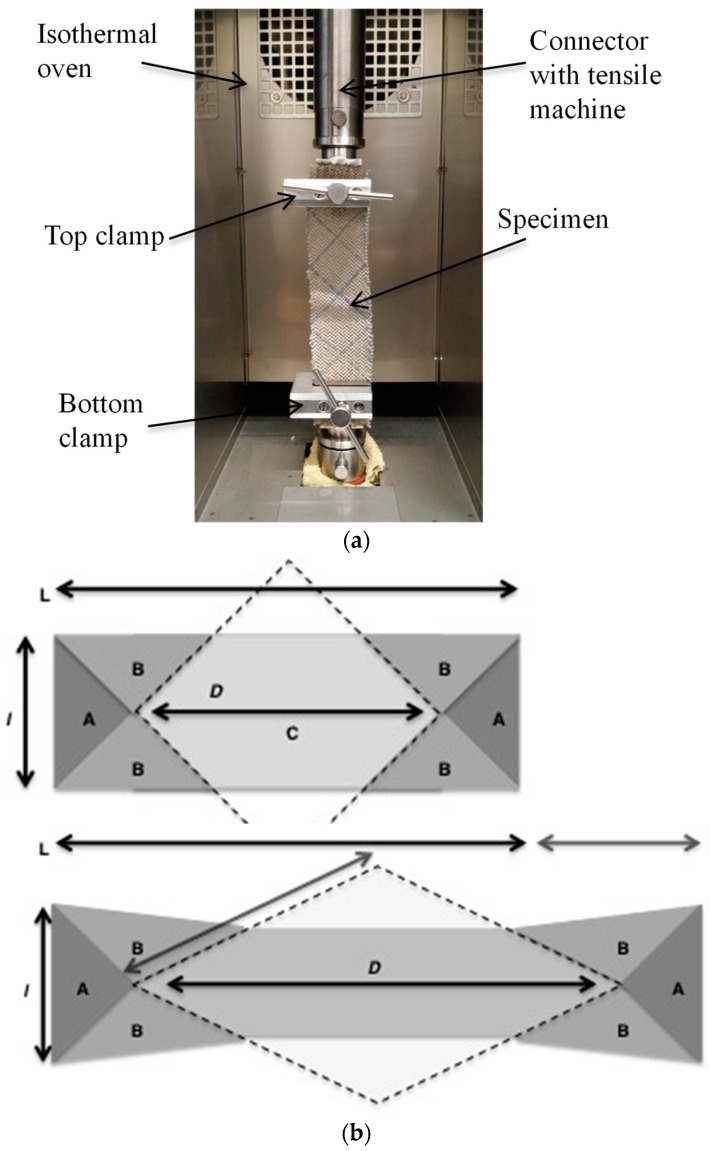
(**a**) Bias extension test set up of the tensile machine with the specimen and (**b**) Undeformed rectangular specimen and deformed shape of the specimen showing half shear and full shear zone.

Figure 3a shows the set-up of the machine for a bias extension test of prepreg made of Flax/PA12. As described in the literature, the bias extension test was performed on the rectangular specimen, which was clamped with warp and weft at a 45° angle relative to the direction of the applied load [16,17,18,19]. To ensure the pure shear zone as shown in Figure 3b(C), the following Equation (1) must stand true.

(1) $$\frac{L}{l}\geq 2$$

where *L* and *l* are the length and width of the specimen, respectively. Thus, the specimen in the present work measured 180 mm × 60 mm.

When the specimen was stretched, the deformation les to in-plane shear deformation; the stages of deformation are shown in Figure 3b. The yarn movement within the specimen generates pure shear due to the yarn in area C being free from the clamp. The angle of the yarn is 90° in the beginning, i.e., where the movement was expected to go through 3 stages; right angle, turning, and change in angle of the yarn (the shear angle before deformation). Contact between fibre resulted in a compression. There is no deformation in zone A, the stretching of the specimen leads to a γ/2 shear in zone B and pure shear in zone C. To calculate the shear angle (γ) with Equation (2), the minimum condition for pure shear must, for the purpose of the stated assumptions, be related to the deformation of the tensile machine [17,20,21,22,23,24,25].

(2) $$\gamma =\frac{\pi }{2}-2\arccos\left(\frac{D+\mu }{\sqrt{2}D}\right)$$

The in-plane shear moment $M_{s}(\gamma )$ relative to the clamping force F(γ) for a given shear angle is shown in Equation (3). Non-linear behaviour was taken into account through an iterative model. Experimental results are computed to generate the material shear behaviour. The function between the in-plane shear moment and the in-plane shear angle is presented in Equation (4) [23,26,27].

(3) $$F(\gamma )\overset{•}{d}=M_{s}(\gamma )\frac{S_{2}}{S_{u}}\overset{•}{\gamma }+M_{s}(\frac{\gamma }{2})\frac{S_{3}}{S_{u}}\frac{\overset{•}{\gamma }}{2}$$

(4) $$M_{s}(\gamma )=\frac{F(\gamma )DS_{u}}{l_{a}(2D-l_{a})}(\cos\frac{\gamma }{2}-\sin\frac{\gamma }{2})-\frac{l_{a}}{2D-l_{a}}M_{s}(\frac{\gamma }{2})$$

where *S*_3_ and *S*_2_ are the original areas of the half shear and full shear zones in Figure 1, *S_u_* is the surface of a unit woven cell in the initial configuration, and $\overset{•}{a}$ is the rate of a quantity *a*. The bias extension tests for prepregs conducted in this paper are shown in Table 3. In this part, the influence of tensile speed on the tensile behaviour of fabric is also tested and discussed; the tensile speed values are shown in Table 3.

All tests for verifying the influence of temperature are conducted within an isothermal oven; temperature was carefully stabilized before the beginning of each test. Each test is conducted more than three times to achieve reproducible and conclusive results.

## 3. Results

### 3.1. Characterizations of a Single Flax/PA12 Yarn

**Figure 4:**
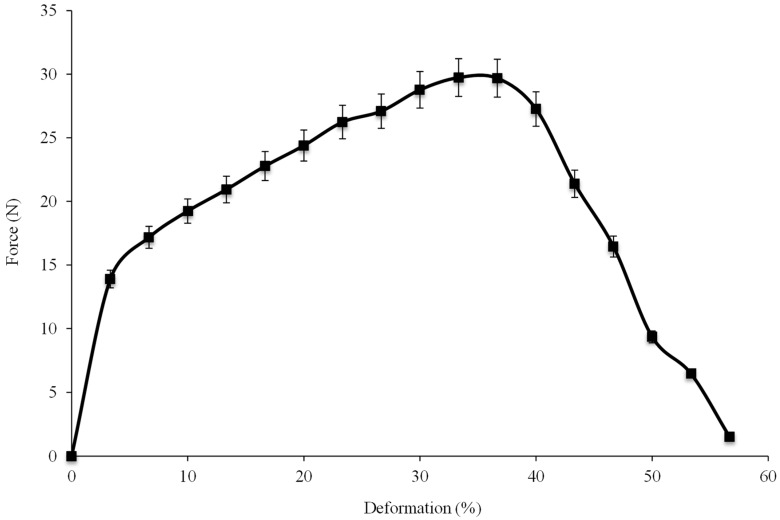
Tensile result of single flax/PA12 yarn.

**Figure 5:**
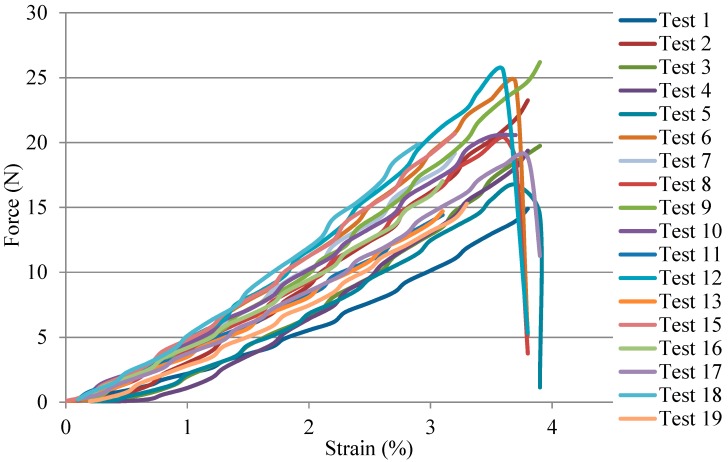
Tensile results of pure flax yarns [28].

The tensile result of a single flax/PA12 yarn at room temperature is shown in Figure 4. Flax/PA12 shows a large deformation with a relatively high force that can be divided into three phases characterised by a rapid increase, a slow increase, and a quick decline. The initial angle of force in phase one is indicative of the force applied to initiate the deformation modulus [3]. The first phase presents a shorter deformation (around 3%) with a steep increase in force, which is similar to the results of pure flax yarn under tensile force, as shown in Figure 5 [28]. In pure flax under tensile force, the small deformation with a rapid argumentation of force was clearly seen before the flax broke. Hence, the first phase in the tensile result of flax/PA12 indicated that the pure flax was broken. But the force required to break the flax is smaller than that for pure flax due to the smaller mass fraction of flax (64%) in flax/PA12 yarn. PA12 also slightly deforms at this phase. However, even though the flax broke at the end of the first phase, the yarn retained strength due to the remaining intact PA12. Hence, a large deformation could be observed in the second phase. The second phase with large deformation and slow slope of increasing force indicated the progressive deformation of yarn (around 3% to 35%). This may be illustrated by a level of slip of PA12 containing the broken flax in the first phase, which is characterised by the tensile behaviour of PA12. The break of PA12 takes place at the end of the second phase. Stepping into the last phase, a steep decrease in deformation after the maximum force is representative of an increase in the progression of a slip of brittle breakage of both the flax and PA12. Nonetheless, not all samples showed brittle breakage of both materials; some specimens generated a buckling movement caused by the irregularities in the yarn due to the flax fibre and its alignment. Yarn reinforcement is created by the alignment of fibres when force is applied. It is well known that fibres provide the highest strength when they are continuous and aligned in the direction of the applied load, resulting in brittle breakages when the strong force is applied. Hence, the irregularity of alignment of shorter flax fibres does not always give rise to brittle breakages, but a buckling movement is sometimes observed.

**Figure 6:**
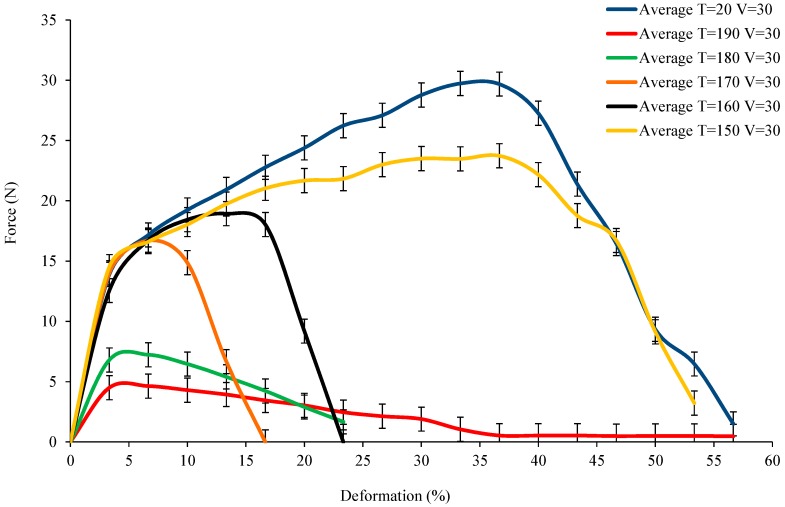
The load vs. deformation for single flax/PA12 yarn under variation of temperature.

The force applied versus the deformation of yarn at different temperatures at 10 °C intervals with a fixed velocity rate of 30 mm/min is shown in Figure 6. The results can be divided into two parts, i.e., below and above the melting temperature. The curves obtained at T = 150 °C, 160 °C, and 170 °C show similar initial increases in force to that of T = 20 °C. The steep increase at the beginning of the curve is indicative of the initial force applied to the extension of the yarn and the slip between the aligned fibres. This means that the yarn deformation/load behaviour is not influenced by the increasing temperature below the melting value in the first phase. However, the increasing temperature has a great influence on the second phase. Dramatically, deformation was shown to decline heavily as temperature increased. This is attributed to the fact that the force required to cause a breakage in PA12 decreases as the temperature approaches the melting value. Hence, the deformation required to reach breakage in PA12 becomes smaller as temperature increases, compared to that of T = 20 °C. In contrast, the T = 190 and T = 180 °C show that the maximum force declines sharply because of the high temperature above the melting value which has the ability to rapidly destroy the PA12 and flax. It is clearly shown that the maximum force at T = 190 and T = 180 °C is also lower than the pure flax tensile force (shown in Figure 5). Hence, the PA12 and flax at the initial phase are both influenced by temperatures above the melting values so that the tension sustaining abilities decrease sharply. However, exceeding the melting temperature can extend deformation at temperatures ranging from 150 to 190 °C. The deformation at 160, 170, and 180 °C was lower than that at 150 and 190 °C. This means that the PA12 under a temperature greater than its melting value (178 °C) is fluid enough to increase the slippage between broken fibres, so that deformation can be prolonged. Comparatively, when the temperature nears the melting value (160, 170, and 180 °C), the PA12 is not fluid enough to change the deformation properties of the yarn. At these relatively lower temperatures, the results show the unreliable mechanical properties of the yarns. It could be speculatively deduced that PA12 impacted by temperatures near its melting value could decrease the mechanical properties of yarn, i.e., decreasing the force in relation to deformation.

**Figure 7:**
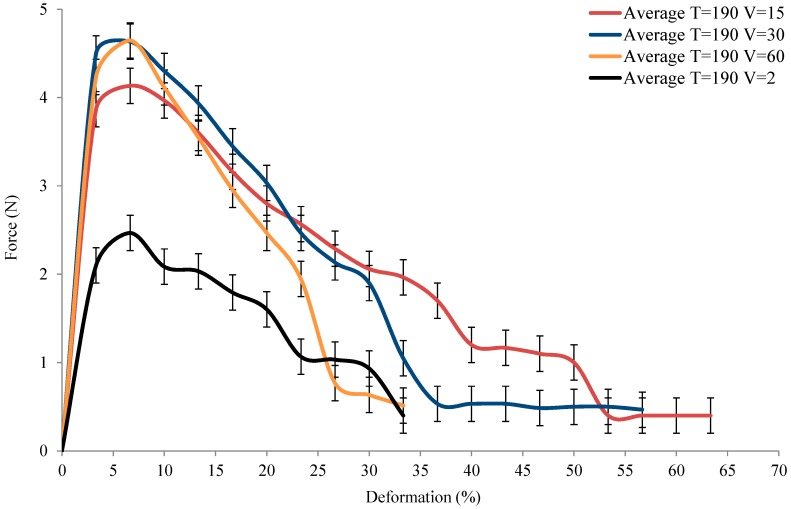
Load vs. deformation under variation of tested speed.

The force applied versus the deformation of yarn at different tensile speeds with a constant temperature T = 190 °C are presented in Figure 7. The obtained deformation curves show a similar sheer increase to T = 20 °C, but the maximum force is considerably lower than at T = 20 °C. A comparison of these results shows that increasing the velocity could increase the force to break the yarn. However, increasing the velocity from 15 to 60 mm/min changes the maximum force only a little. This is probably because the intervals of change in velocity are too small to present an influential analysis of how different velocities can alter the Flax/PA12’s tensile behaviour. In contrast, the maximum force at V = 2 mm/min is about half that at V = 60 mm/min, which is due to the fact that the temperature has enough time to change the properties of flax and PA12 so that the ability to sustain the force decreases sharply. It is also noted among the results of increasing velocity from 15 to 60 mm/min that a lower velocity has a longer the extension of deformation, i.e., the higher the maximum force, the steeper the loss of force relative to deformation. Hence, it is inferred that the velocity can change the tensile behaviour at relatively low velocities (2 mm/min), and that high velocities (60 mm/min) both yields the small deformations.

### 3.2. Characterizations of 2 × 2 Twill Woven Fabric with Flax/PA12 Yarns

**Figure 8:**
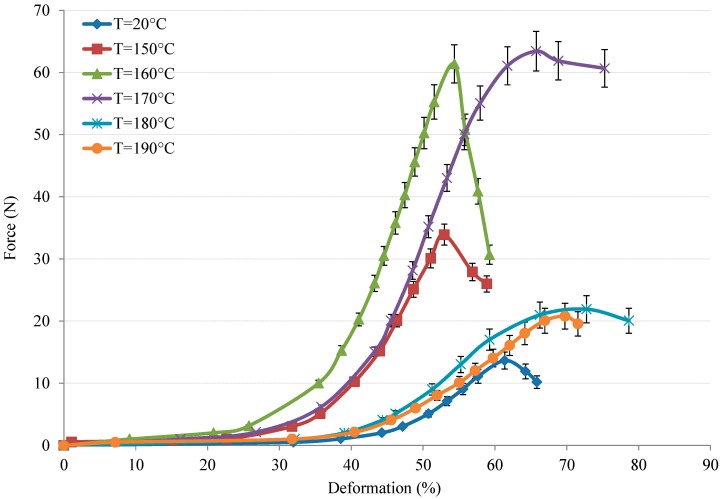
The load vs. deformation under variation of temperature.

The in-plane result of fabrics is shown in Figure 8 at intervals of 10 °C with a fixed velocity of 30 mm/min. In Figure 8, the curves show tests conducted below and above the melting temperature, which presents a similar evolution—i.e., one that can be divided into three main phases—as that ignoring the different temperatures. At first, a weak load is necessary to overcome the adhesive friction effect between the yarns to start intra-ply shearing. Then, the in-plane shear increases under moderate forces. However, when the yarns begin to contact with each other in zone C, the load increases noticeably to the maximum. Finally, the load decreases sharply because of fabric fracture. Although the results under different temperatures show a similar evolution of displacement vs. force, the values of thermo-mechanical behaviour strongly depend on temperature, since the maximum load at different temperatures has complicated results. The maximum load increases with increasing temperature below the melting value. Beyond the melting value, the maximum load decreases sharply. However, all the maximum loads at different temperatures are greater than that at room temperature (T = 20 °C), which presents the inverse trend compared to that of a single yarn (see Figure 6). This is indicative of the strength being enhanced by increasing temperature. This is because PA12 is not mobile enough, so friction increases during lateral contact (second phase); thus, the maximum load increases as the temperature approaches the melting value of PA12. When the tested temperature exceeded the melting value of PA12, the PA12 is melted, i.e., in liquid form, so that the friction is almost not existence. Hence, the maximum load decreases dramatically, as shown in Figure 8. Therefore, it can be concluded that increasing the temperature could enhance the mechanical behaviour of Flax/PA12 woven fabric.

**Figure 9:**
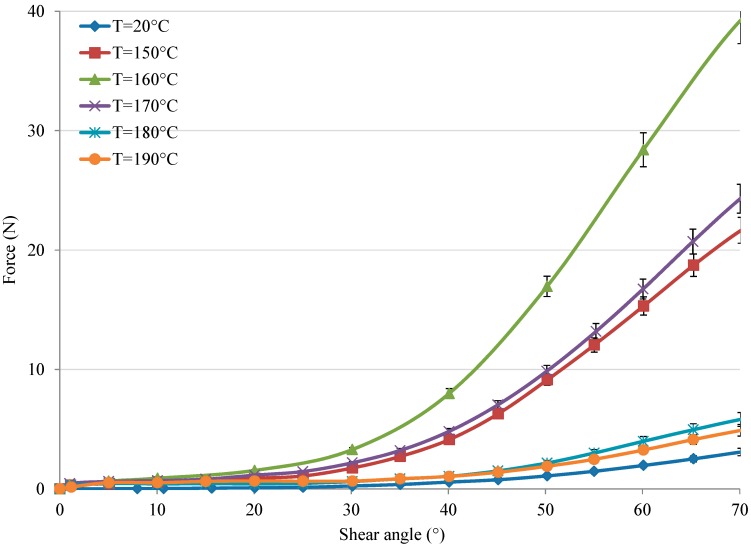
The shear angle vs. load under variation of temperature.

Analysing the characteristics in the relationship between the shear angle and deformation, as seen below in Figure 9, can identify the influence of the thermo-mechanical behaviour through the shear angle when the specimen is under different temperatures. It is observed that an increase in temperature causes an increase in the force required to reach the maximum shear angle when the temperature is below melting point. When the temperature is above the melting point of PA12, the force needed to reach the maximum shear angle drops dramatically, since the properties of PA12 change a lot. It was further confirmed that the strength could be increased with increasing temperature below the melting value. However, the angle cannot be calculated past 90° because of the deformation caused by the slip and compression of the interlacing yarns.

**Figure 10:**
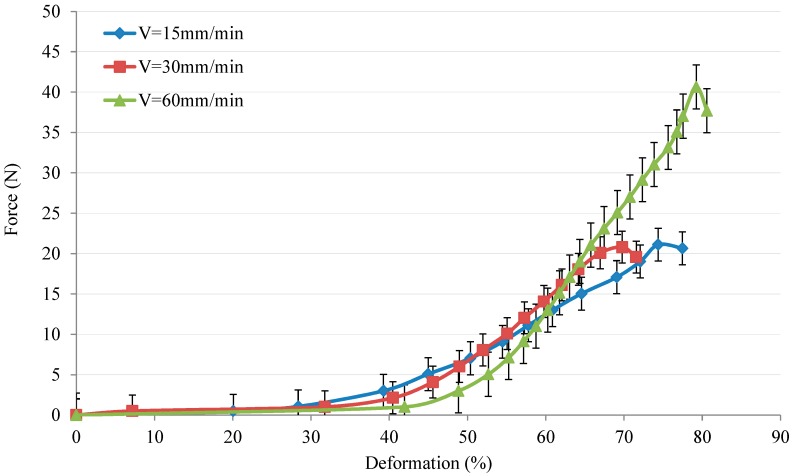
The load vs. deformation under variation of tensile speed at the fixed temperature.

The in-plane behaviour for flax/PA12 fabric under different tensile speeds over the PA melting temperature (at 190 °C) is shown in Figure 10. The result for three tensile speeds shows that the load/displacement under 15 mm/min and 30 mm/min have a similar trend, i.e., with nearly the same maximum loads. Nonetheless, the result under 60 mm/min shows that the strength increased, since the maximum load under 60 mm/min is about double that of 15 mm/min and 30 mm/min. That signifies that the property of tensile behaviour improved by increasing the tensile speed, because of the PA melting without enough time. Thus, the fabric strength remains relatively high in terms of its ability to resist tension. This result is also nearly identical with single yarn experiments. However, almost the same results were found under 15 mm/min and 30 mm/min; this is possibly due to the fact that the interval is a little bit small, which could not significantly influence the result.

**Figure 11:**
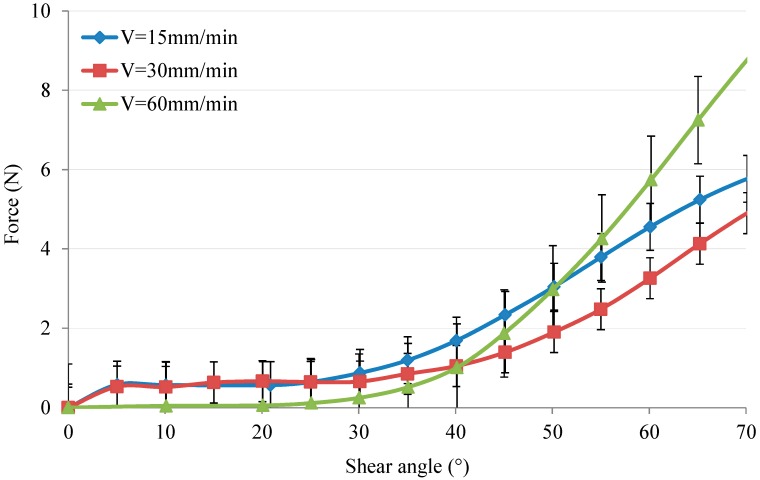
The shear angle vs. load under variation of speed.

The shear angle characterisation under different tensile speeds at 190 °C is shown in Figure 11. It is clearly observed that the evolution of shear angle by 60 mm/min needs the bigger effort than by 15 mm/min and 30 mm/min, which also shows that strength could be influenced by variations in tensile speed. Meanwhile, the tensile speed should be increased relatively so that the strength can be changed. Otherwise, the strength is almost not affected with speed increasing from 15 mm/min to 30 mm/min, as shown in Figure 11.

## 4. Conclusions

In this paper, the thermo-mechanical characteristics of the tensile and in-plane shear properties of single Flax/PA12 yarn and 2 × 2 Twill woven Flax/PA12 fabric are presented. The data were recorded under a range of temperatures and tensile speeds during the thermoforming process, and display the differences in the tensile performance of Flax/PA12 both in single yarn and fabric forms. The Flax/PA12 single yarn mainly experienced two phases at room temperature, consisting of pure flax yarn which broke with relatively low deformation during the first phase, and PA12, which broke with large deformation during the second phase. However, increasing temperature during tension tests, the deformation decreased markedly in the second phase. Over the melting value of PA12, the strength declines dramatically. Comparatively, the strength can be improved by increasing the tensile speed when the temperature is beyond the melting value of PA12.

A comparison of the thermo-characteristics of woven fabrics, however, shows that fabric strength increases with increasing temperature, especially when the tested temperature approaches the melting value of PA12. Beyond this value, the strength decreases sharply but is still a little bit higher than that of room temperature tests. The tensile speed has a similar effect on the in-plane results of woven fabric as on single yarn, in which increasing temperature could enhance the fabric strength.

In the future, it will be necessary to analyse the bending properties of the Flax/PA12 to determine how the shear angle is affected whilst ensuring that the mechanical characteristics are not compromised.

## Figures and Tables

**Table 1:** The main properties of the Flax/PA12 commingled yarn and woven fabric.

	Parameters	Values
Flax/PA12 commingled yarn	Manufacturer	Schappe Techniques
	Mass fraction	Flax (64%)/Polyamide 12 (36%)
	Flax fibre diameter	25 µm
	PA12 fibre diameter	12 µm
	Linear density (tex)	500
Flax/PA12 woven fabric	Type of fabric	Twill 2-2
	Manufacture	Schappe Techniques
	Yarns (warp/weft)	500 tex/500 tex
	Area density (g/m^2^)	380 ± 5
	Thickness (mm)	1.84
	Number of warp yarns per cm	3.8

**Table 2:** The tensile test condition for a single yarn thread.

	T (°C)	V (mm/min)
Room temperature and extension	20	30
Variants of T with fixed V	150	30
	160	
	170	
	180	
	190	
Variants of V with fixed T	190	2
		15
		30
		60

**Table 3:** The bias extension test condition.

	T (°C)	V (mm/min)
Room temperature and extension	20	30
Variants of T with fixed V	150	30
	160	
	170	
	180	
	190	
Variants of V with fixed T	190	15
		30
		60
